# On stance-taking with one-sided vs. two-sided shoulder lifts in German talk-in-interaction

**DOI:** 10.3389/fpsyg.2025.1509988

**Published:** 2025-04-28

**Authors:** Emma Betz, Alexandra Gubina

**Affiliations:** ^1^Department of Germanic and Slavic Studies, University of Waterloo, Waterloo, ON, Canada; ^2^Leibniz Institute for the German Language, Mannheim, Germany

**Keywords:** shrug, shoulder lift, embodiment, stance, accountability, responsibility, resistance, multimodal Conversation Analysis

## Abstract

Taking a stance toward events, objects, and other persons is fundamental to human interaction. We investigate one specific body movement that is involved in stance-taking in interaction: a shoulder lift, realized as either a one-sided or a two-sided movement. Using multimodal Conversation Analysis, we trace how interactants employ shoulder lifts in different positions within responsive turns in various interaction types in German. This study reveals how the actions to which shoulder lifts contribute are bound to specific turn and sequence positions. We demonstrate how shoulder lifts are used for disclaiming the speaker's accountability or responsibility by framing their turn as non-expandable or non-expansion-worthy, thus curtailing the sequence. Furthermore, the study shows how participants orient to different types of shoulder movements, i.e., lifts with one or with both shoulders, as accomplishing different interactional tasks. By showing that shoulder lifts are a positionally sensitive resource for speakers in building stances, we showcase the potential of conversation analytic and interactional linguistic approaches to further our understanding of multimodal stance-taking in interaction.

## 1 Introduction

Taking a stance toward events, objects, and other persons is fundamental to human interaction. Stance-taking can foreground matters of knowledge, rights/responsibilities to act, and affect (e.g., Du Bois, 2007; Heritage, 2012; Goodwin and Alim, 2010; Stevanovic and Peräkylä, 2014). Stance-taking involves resources of language and body. Prior interactional research has concentrated on linguistic practices for stance-taking; work on embodied practices has started to emerge recently (e.g., Cekaite, 2010; Clift, 2024; Ford et al., 2012; Heller et al., 2023; Kaukomaa et al., 2014; Marrese et al., 2021). Our paper contributes to this latest line of research by investigating a specific body movement that is involved in stance-taking in interaction—a shoulder lift, realized as either a one-sided or a two-sided movement. Using multimodal Conversation Analysis (CA; Sidnell et al., 2012; Mondada, 2018), we trace how interactants employ shoulder lifts in different positions within responsive turns in a range of interaction types in German.

Prior research has described shoulder lifts (commonly understood to involve both shoulders) as one core component of “shrugs” (Debras, 2017; Givens, 1977; Morris, 1994; Streeck, 2009)—complex ensembles including such elements as head tilts, shoulder lifts, and certain mouth configurations. It has been suggested that the “shrug” has a more general unified meaning (*disengagement*, Streeck, 2009, pp. 189–91; cf. Debras and Cienki, 2012), and that this can convey distinctly different stances: incapacity and non-responsibility, affective distance or indifference, epistemic meanings like indetermination and common ground (Debras, 2017).

The present study heeds the call for more research on the body movements commonly associated with “shrugs” in different languages and in a broader range of data, specifically in spontaneous, unguided, naturally occurring interactions. We analyze shoulder lifts in their precise sequential environments and ask (a) where/how speakers use different types of shoulder lifts (i.e., one-sided vs. two-sided) systematically in real-time interaction, (b) what actions lifts contribute to and what stances they (contribute to) convey(ing), and (c) whether there are differences in use between different types of lifts in different positions within responsive turns.

The following data extract includes three separate shoulder lifts and offers a glimpse of the range of our phenomenon. It comes from a mealtime interaction between friends Gero, Zoe (seated to Gero's left), and Zoe's boyfriend Norbert (seated across from Gero). In lines 01–02, Gero initiates a new topic and sequence by announcing that a new intern has joined their department. In line 3, Norbert first acknowledges the news and then pursues assessment by Gero with a rising-intoned *und* (“and”). In response, Gero first produces the vocalization *pf* (line 5; see Baldauf-Quilliatre and Imo, 2020) that projects a negative assessment, which is uttered in line 8. Norbert responds by laughing, and Zoe criticizes Gero for always judging “them” (meaning either female interns or women in general) based on their outward appearance (lines 10–11).

In response, Gero produces three different types of shoulder lifts:

- A two-sided movement accompanying his account in line 13 (Figures 1–3),
- a two-sided movement after catching Zoe's gaze in line 15 (Figures 4–6),
- and a one-sided movement with his concession in line 16 (Figures 7–9).

**Figure:**
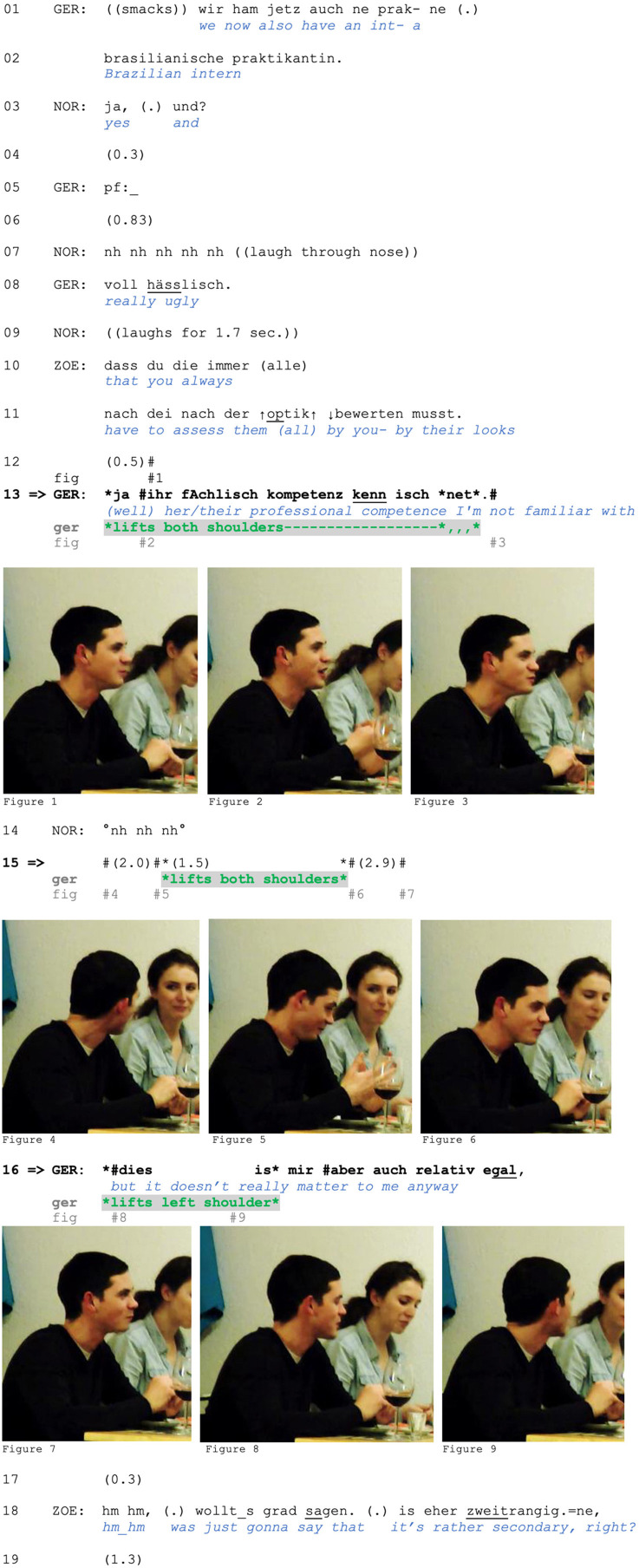
**Extract (1): FOLK_E_00293_SE_01_T_04_DF_01_c913_zweitrangig** http://bit.ly/3BS40hu

Example 1 shows that shoulder lifts occur in different turn positions: in responsive position accompanying talk, before a possible turn completion, in “pre-beginning” position, and in “post-possible completion” position (Schegloff, 1996a, pp. 90–93). In some cases, shoulder lifts can be produced not only within responsive turns but as *responsive turns per se*. In this paper, we show that shoulder lifts in these different positions accomplish various kinds of interactional functions, but we also reveal that they share commonalities: In all these cases, shoulder lifts are used for

- disclaiming the speaker's accountability, or responsibility (in the case of Extract 1 for their own behavior) through
- framing their turn as non-expandable, or non-expansion-worthy, and thus
- curtailing the sequence.

Furthermore, we will show that the interactional work of a shoulder lift and thus its meaning depend not only on where it occurs within a TCU/turn (position), but also how it is produced (composition), i.e., as a one-sided or two-sided lift. Specifically, this study will demonstrate that movements with one or with both shoulders are employed for—and afford for—accomplishing different interactional work across various settings and sequential positions: While two-sided shoulder lifts are systematically employed for indexing the speakers' lack of *ability* to “go along,” or to further engage with the Other's initiated or projectable course of action (e.g., due to lack of knowledge), one-sided shoulder lifts tend to mark “non-engagement,” in other words, *resistance* to “go along” with the Other's initiated or projectable course of action.

## 2 Paralinguistic and bodily resources for stance-taking in talk-in-interaction

Taking a stance—be it toward a material object, a person, or a turn-at-talk—is omnirelevant in talk-in-interaction, i.e., “there is never a time out from the social action of taking stances and adopting positions” (Du Bois and Kärkkäinen, 2012, p. 438). By taking a stance, participants publicly position themselves toward a stance object through evaluating it. In doing so, they either align or misalign with co-interactants' stances (Du Bois, 2007; Du Bois and Kärkkäinen, 2012). Most studies within Conversation Analysis and Interactional Linguistics on stance-taking (see Stevanovic and Peräkylä, 2014) concentrate on *linguistic* means for stance-taking. However, other resources for taking a stance in talk-in-interaction have received increasing attention across different languages (see Andries et al., 2023 for a comprehensive overview).^1^

Stance-taking can be accomplished with paralinguistic means, which include non-lexical vocalizations, or sound objects, i.e., “conversational objects with [phonetic substance but] minimal semantic content.” These include “so-called ‘primary interjections,' such as, e.g., *oh, ah* and *ooh*, and non-lexical sounds such as clicks and whistling, which have been found to function similarly in talk-in-interaction” (Reber, 2012, p. 12; Keevallik and Ogden, 2020). Some of these “sound objects” are strongly associated with displaying certain emotions and accomplishing affective work, such as expressing surprise (Wilkinson and Kitzinger, 2006) or disappointment (Couper-Kuhlen, 2009), expressing “suffering” with moaning, whining or crying (Edwards, 2005; Hepburn and Potter, 2007), conveying a sense of pleasure with gustatory *mmm* (Wiggins, 2002; Golato, 2011), or conveying disgust with *eugh* (Wiggins, 2013). Interactants can also position a specific referent as laughable with laughter (Jefferson et al., 1977; Glenn and Holt, 2013) or convey a negative stance toward an assessment with the “sound object” *pf* (see Extract 1, line 5), thus indicating that they lack the words to verbally express the assessment in a different way (Baldauf-Quilliatre and Imo, 2020, p. 212).

Another group of non-lexical vocalizations that can display affective stance are respiratory phenomena like whistles, sighs, and clicks. In their study on melodic and non-melodic whistles, Reber and Couper-Kuhlen (2020) demonstrate that a non-melodic gliding whistle that “begins on a high tone and glides slowly downwards” (p. 177) systematically occurs in response to informings that describe a norm-breaching state of affairs and often contains a numerical reference. The gliding whistle conveys a specific affective stance toward the prior informing: It treats it as impressive and/or shocking (see also Reber, 2012, ch. 9.2). Clicks constitute another interactional resource that is used for stance management in talk-in-interaction (Reber, 2012, ch. 9.1; Wright, 2011; Ogden, 2013, 2020). Reber demonstrates that clicks produced in response to complaints can index “disapproval” of the complainable and thus display “a concordant and hence affiliative stance, yet without bringing in too much affective involvement” (Reber, 2012, p. 234). Ogden (2020) shows how clicks are used as a practice for audibly not accomplishing socially improper actions (like self-praise or sexual allusions) that could “put the (non-)speaker in an awkward or conflictual position” (Ogden, 2020, p. 86). Sniffing can project dispreferred responses or be part of “delicate” actions (Hoey, 2020). Sighing is routinely and indexically associated with negative affect and can thus project a negative valence (Hoey, 2014).

Taking or projecting a stance can also be accomplished through bodily means. These include facial expressions (Groß et al., 2023). Turn-beginning frowns in particular have been shown to anticipate turns that are potentially problematic and can constitute a complication, like an upcoming negative assessment, disaffiliation, or epistemic difficulty (Kaukomaa et al., 2014). Turn-opening smiles can project a “shift from a neutral or serious emotional stance to a positive or humorous emotional stance” (Kaukomaa et al., 2013, p. 21). And an eyeroll can display protest or dissent with the prior speakers' actions, as Clift (2021) demonstrates.

Recent studies have also focused on how multimodal packages - combinations of verbal, vocal, and bodily resources - can display specific stances. Clift (2023) demonstrates how “raising of the eyebrows—which has the effect of furrowing the brow—in conjunction with a pursing of the lips” (Clift, 2023, p. 172) can be treated as a display of skepticism. Similar results were obtained by Heller et al. (2023), who show how bodily, verbal, and prosodic resources are employed in tandem to display a critical stance in two actions: contradicting another and calling something into question. Contradicting is characterized by contracting and lowering eyebrows and by narrowing of the eyes, gaze aversion from the recipient, and nose-wrinkling. Questioning, by contrast, consistently occurs with prolonged contractions and an upward or downward movement of the eyebrows, as well as direct gaze at recipients. Depending on whether the eyes are narrowed or wide and depending on which specific prosodic resources are employed, the stance displayed through questioning can be more confrontational or less so.

Stance displays can also be accomplished with embodied resources other than facial expressions. Clift (2014) describes a practice called “visible deflation,” which is characterized by “a bodily ‘let-down' from a position in tension” (Clift, 2014, p. 381) and used for displaying a negative stance—“exasperation”—in response to others' actions. In a study on complaints in French, Skogmyr Marian (2021) demonstrates that specific types of embodied conduct can be used to complete incomplete verbal turns and display a negative affective stance. Such resources encompass depictive gestures (Streeck, 2009) that have a conventional negative meaning, embodied gestures associated with expressing negation and negative stance (Kendon, 2002), or pragmatic gestures (Kendon, 2004) such as the placing of hands on the table. Our article continues this line of research by focusing on shoulder lifts and their role in stance-taking in social interaction.

## 3 Shoulder lifts in interaction

As noted in Section 1, prior research has treated shoulder lifts as the most prototypical, core component of shrugs (Streeck, 2009; Debras and Cienki, 2012), which is why “shoulder lift” and “shrug” are often used as synonymous descriptions. Earlier studies on shrugs have classified them as “emblems” (Ekman and Friesen, 1969) or “quotable gestures” (Kendon, 2004, p. 335), i.e., as gestures with a fixed, conventionalized meaning within a specific culture. In contrast, recent, more interactionally-grounded approaches have treated shrug as a complex enactment, in which “a single component or a combination of components can index the whole enactment” (Debras, 2017, p. 1; Streeck, 2009, p. 190). Possible components include raised eyebrows, palm-up gestures, head tilts, mouth shrugs (i.e., when the corners of the mouth turn downward and the middle part of the mouth is raised, see Extract 5, Figure 14 for an example), possibly in combination with an upward-forward chin movement and shoulder lifts. However, it is important to note that single components can vary in the degree of specificity of their meaning, e.g., a mouth shrug has been associated with the specific meaning of epistemic negation or ignorance (Givens, 1977, p. 13; Morris, 1994, p. 65; Streeck, 2009, p. 190), while shoulder lifts can accomplish other functions, as we will show in Section 5.

Prior research has also proposed a general, or context-free, meaning of shrugs, namely *disengagement* (Kendon, 2004, p. 265; Streeck, 2009, p. 190; Debras, 2017) or “*dis-stance*”: Speakers position themselves “with respect to a prior stance, while simultaneously acknowledging [a] stance differential” (Debras and Cienki, 2012, p. 6; see Du Bois, 2007) between their positioning and that of other interlocutors (see also Schegloff, 1996a, p. 92, who lists shrugs as stance markers that can appear at possible turn completion). This general meaning has also been specified as displaying a participant's ignorance or uncertainty (Tennant and Brown, 1998, p. 180), non-intervention to whatever is in focus (Kendon, 2004, p. 275), or non-assertiveness (Givens, 1977), and as functioning as a disclaimer (Morris, 1994 on facial shrugs).

More interaction-oriented approaches (Debras, 2017, p. 12) differentiate between three semantic categories, or meanings, of shrugs: (1) incapacity and non-responsibility, (2) affective distance or indifference, (3) epistemic meanings like expressing indetermination and indexing common ground. Shrugs have also been shown to accomplish functions similar to epistemic-evidential markers, “which relate[...] to the gesturer's degree of knowledge of, and commitment to, a state of affairs, and to the origin of a gesturer's knowledge” (Debras and Cienki, 2012, p. 936). For instance, shoulder shrugs (together with direct gaze on recipients) co-occur with formats like *je (ne) sais pas* (Debras, 2021; Pekarek Doehler, 2022), indexing lack of epistemic access in response to questions. Further functions in question are uncertainty, obviousness (see Jehoul et al., 2017; Schoonjans, 2018, pp. 160–64), and logical or chronological necessity.

As this overview demonstrates, prior research does not systematically differentiate between shrugs as complex configurations and shoulder lifts as one particular resource with possibly distinct features (e.g., one-sidedness) used for accomplishing specific interactional work. Debras (2017, p. 17) acknowledges a possible perceptual difference between one-sided and two-sided shoulder lifts by describing one-sided shrugs as “less conspicuous,” but it remains unclear whether participants actually orient differently to one- and two-sided shoulder shrugs (i.e., as accomplishing different interactional tasks). Moreover, interactionally oriented research on shoulder shrugs is based primarily on semi-guided conversations (Debras, 2017; Debras and Cienki, 2012), which is why we still know little about how and when shoulder lifts appear in naturally-occurring interaction. The present study addresses these open questions by systematically investigating the use of one-sided and two-sided shoulder lifts in naturally occurring, unelicited and unguided interactions in German. We seek to determine how different types of shoulder lifts are employed by participants in different positions within turns and sequences to accomplish, or contribute to, distinct interactional functions. Moreover, we will demonstrate that shoulder lifts are different from most of the para- and non-verbal resources used for stance-taking already described (see Section 2) in terms of their *stance object*: In contrast to other non-verbal resources that can be used for taking a stance, shoulder lifts allow speakers to position themselves not toward *what another participant said or did*, but rather toward *their own utterance(s)*. By analyzing a diverse set of interaction data, we explore the nuanced roles that shoulder lifts play in stance-taking and in talk-in-interaction in general.

## 4 Data and methods

Research on multimodal interaction has shown that the precise temporal ordering of embodied behavior and talk is constitutive for social action. Using multimodal CA (Mondada, 2018), we trace how interactants employ shoulder lifts in different turn positions in a range of interaction types in German. Our data come from the German FOLK corpus^2^ and are drawn from more than 80 h of video recordings of naturally occurring face-to-face interaction in everyday and institutional contexts (e.g., driving lessons, physiotherapy sessions) in both stationary settings (e.g., family breakfasts, playing table-top games) and mobile configurations (e.g., joint activities such as cooking or renovating a room). A small number of additional examples were added as we came across them while working on other projects; these are from private corpora (including mundane and workplace interaction, face-to-face and video-mediated) and from public interaction (e.g., political debates). All non-public data were collected with participants' informed consent, and all names appearing in the transcripts have been anonymized.

The initial collection we assembled consisted of 259 cases, of which 108 were identifiable as two-sided shoulder lifts and 118 were one-sided lifts. In 33 cases, we could—due to the camera angle, the quality of the recording, or the spatial arrangement of participants—not determine with confidence whether one or two shoulders were lifted, or whether we were dealing with a communicative shoulder lift at all (as opposed to a shoulder movement that resulted from adjusting a different part of the body or from the body's involvement in laughter). These examples were set aside. CA methodology (Schegloff, 2007; Sidnell et al., 2012; Robinson et al., 2024) allowed us to relate shoulder lifts to the precise turn and sequential positions in which they occur in the back and forth of real-time interaction. Since we were interested in participants' orientations to the actions and positionings accomplished by turns that consisted of/contained shoulder lifts (on participant orientations, see Raymond and Robinson, 2024), we limited our focus to broadly responsive uses. These include shoulder lifts that stand alone *as* responses, those that accompany responsive verbal turns, and those that are produced at the boundaries of responsive turns (i.e., shoulder lifts that precede verbal turns, complete them, or follow possibly complete verbal turns). We did not limit our focus to specific action environments in which shoulder lifts occur. However, most of the shoulder lifts in our collection are produced in response to informings; requests for information or confirmation; as well as challenges, reproaches, and account solicitations. Due to space limitations, we excluded shoulder lifts produced as part of multi-unit turns (e.g., story-tellings, 139 instances).^3^ The resulting collection of target cases for this study contained 87 instances (44 one-sided and 43 two-sided shoulder lifts).

The examples assembled in this collection were examined individually in detail—including the shape of the shoulder lifts and their precise placement in turn, sequence, and larger activity—and compared to each other to uncover commonalities in formal, sequential, and action patterns (on working with collections, see Clayman, 2024). In an iterative process, we were able to identify distinct contexts of use and stable ways in which shoulder lifts contribute to stance-taking in interaction. We analyzed around 50 cases in this way before reaching a point of analytic saturation (Corbin and Strauss, 1990). We then checked each of the remaining examples in the collection to test and refine our findings and to determine the breadth and variability of the practice.

In Section 5, we report our findings and show representative examples of the different uses of shoulder lifts we uncovered in spontaneous interaction. The extracts were transcribed according to Jeffersonian and Mondada transcription conventions (Jefferson, 2004; Mondada, 2024), and talk is presented in the original German with idiomatic translation into English. The shoulder lifts are highlighted in gray and shown in green font. Responses that are accompanied, preceded, or followed by shoulder lifts are marked with an arrow (= >) on the left. Each extract is accompanied by a link to the video data (see Footnote 2 for corpus access details). In inspecting the data, readers will notice that shoulder movements are quite prominent in some instances (e.g., Extracts 11 *dahergesetzt*, 13 *nervich*) and small and initially hard to see in others (e.g., Extract 2 *sixt*). Furthermore, some movements we classified as one-sided may not initially appear to be one-sided. This is because in all shoulder movements, other parts of the upper body are invariable involved, so that one can observe slight movement of the other shoulder in many one-sided shoulder lifts. In analyzing our examples, we used differences in the prominence of movement to determine whether we are dealing with two- or one-sided lifts. A one-sided shoulder lift in our collection is one in which the movement of (only) one side seems deliberate and is more prominent.

## 5 Shoulder lifts in different turn positions in interaction

This section is organized to represent different positions in which shoulder lifts systematically occur relative to the verbal responsive turn. We thereby illustrate how the actions to which shoulder lifts contribute are bound to (and derive their meaning from) specific turn positions. This is crucial for the different kinds of stance-taking that shoulder lifts can accomplish. The uses we show include shoulder lifts in pre-beginnings of responses (Section 5.1), lifts accompanying verbal responses (Section 5.2), lifts used before—and alternative to—projected turn completion (Section 5.3), lifts after a possible turn completion (Section 5.4), and shoulder lifts as stand-alone responses (Section 5.5). We present examples of both one-sided and two-sided shoulder lifts to build our argument that these two types of lifts have different affordances when it comes to stance-taking. By showing that shoulder lifts are a positionally sensitive resource for speakers in projecting, building, and retrospectively showing stances, we also hope to showcase the unique potential of conversation analytic and interactional linguistic approaches to further our understanding of multimodal stance-taking in interaction.

### 5.1 Shoulder lifts in pre-beginnings

We begin our analysis with shoulder lifts that occur in pre-beginnings, i.e., before a responsive turn's recognizable beginning. In this position, speakers use shoulder lifts to *project* the stance a responding speaker will take *before* they start of a verbal turn—a function that other non-verbal resources, such as frowns, smiles (Schegloff, 1996a; Kaukomaa et al., 2014), or sighing (Hoey, 2014; Schegloff, 1996a, pp. 105–106; see Section 2) can also accomplish.

Extract (2) is from an interaction between Isabell (ISA) and Ferdinand (FER) during a car ride. Before the extract, the participants had started talking about different rental care companies. Isabell now notes that two companies are represented throughout Europe and that she believes that one of them, SIXT, was founded in Germany. In line 2, she initiates a telling to support this belief: she saw a report about the wealthiest families on a TV channel (*RTL*) and the owners of SIXT were among them (lines 2–7). After a minimal acknowledgment by Ferdinand in line 9, Isabell reiterates that they belong to the richest families in Germany (line 11–12). She then downgrades her certainty regarding their country of origin (line 12), and in line 14, she settles on Germany as likely correct. After a 1.7-s pause, in which no reaction from Ferdinand is forthcoming, she starts moving her gaze toward Ferdinand (line 15).

**Figure:**
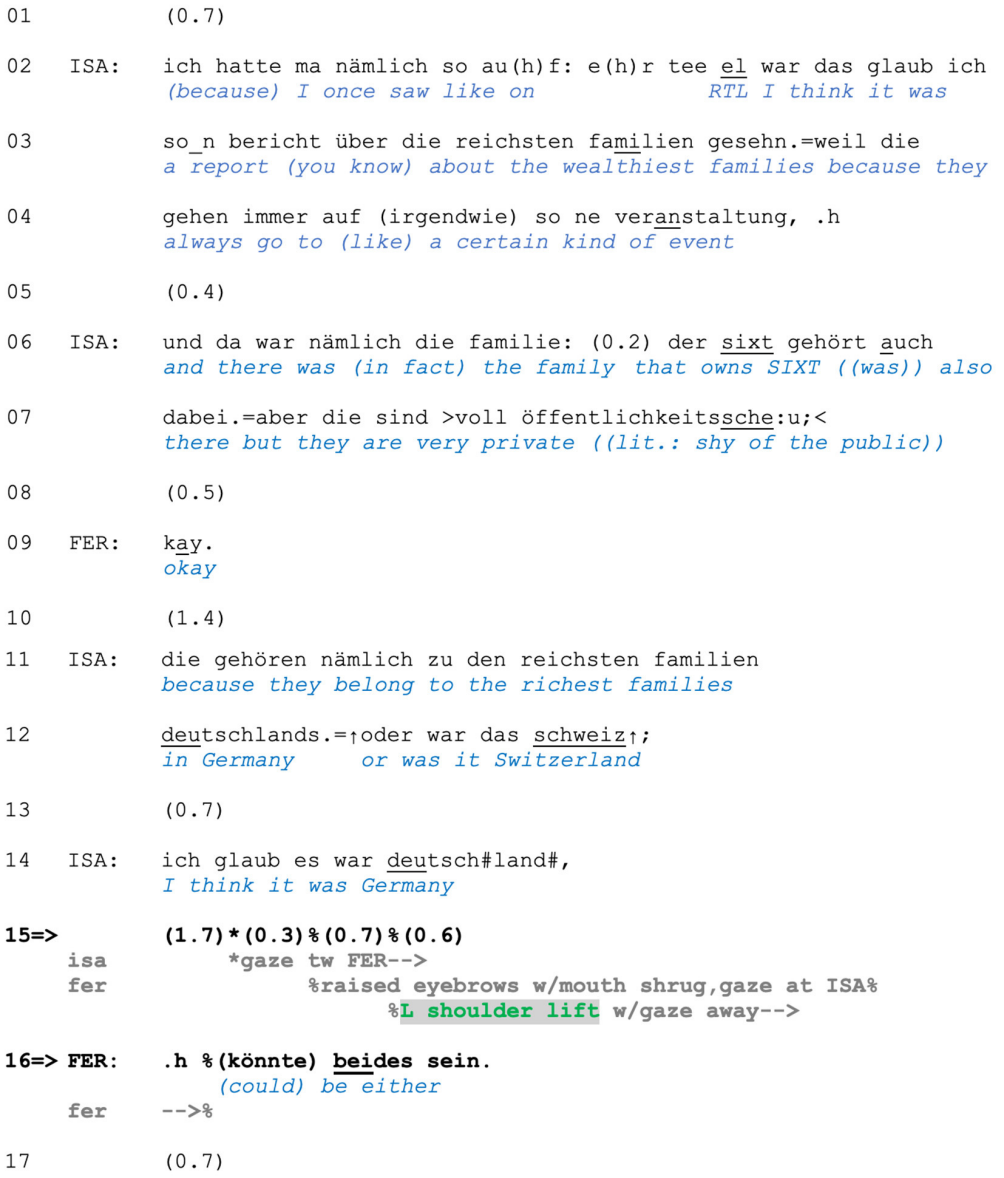
**Extract (2): FOLK_E_00301_SE_01_T_01_DF_01_sixt** https://bit.ly/3BJlg8P

Although Isabell displays doubt regarding her knowledge of the country of origin of the company's founders (lines 12, 14) and thus demonstrate s trouble with remembering, Ferdinand does not produce any responses. This may be why he seems to interpret Isabell's gaze toward him (line 15) as mobilizing a (lacking) response (Stivers and Rossano, 2010). As soon as Isabell starts turning her head and gaze toward him, Ferdinand raises his eyebrows, turns down the corners of his mouth, gazes directly at Isabell, and produces a left-shoulder lift right before suggesting that either country (Germany or Switzerland) is plausible.

In neither confirming nor disconfirming Isabell's assumption, Ferdinand produces a non-answer (Stivers, 2022, Ch. 3), which displays not only a lack of knowledge but also a certain degree of uncooperativeness by not telling what he thinks is likely. With his verbal and non-verbal behavior, Ferdinand observably refuses to engage in further reasoning about the matter in question and thus with the activity initiated by Isabell's trouble in remembering. Such one-sided shoulder lifts project a response that does not “go along” with the terms, expectations, or agenda set up by the prior speaker's turn, and they thus mark the speaker's resistance to engage with the Other's projectable response expectations, topical and sequential development, and the epistemic positioning expected from them. In doing so, responding speakers mark their response as non-expandable and propose sequence closing.

Non-answers, or misaligned responses, are not the only type of uncooperative responses projected by shoulder lifts in our data. The following extract, taken from a conversation between three friends, shows how a one-sided shoulder lift can be used for projecting a *disaffiliative* response. Melissa has just said that she and her partner were considering moving in together and that she can imagine taking that step (lines 1–2). Saskia responds by expressing doubt about living together with her own partner, Thomas (lines 4–8) and then accounts for this by anticipating a specific challenge (lines 10–14), namely that her *sparsamkeitsverhalten* (“way of saving money;” line 14) may differ from his and that this may lead to friction. After a pause, Melissa produces a one-sided shoulder lift (line 15) and then a verbal response (line 16–19).

**Figure:**
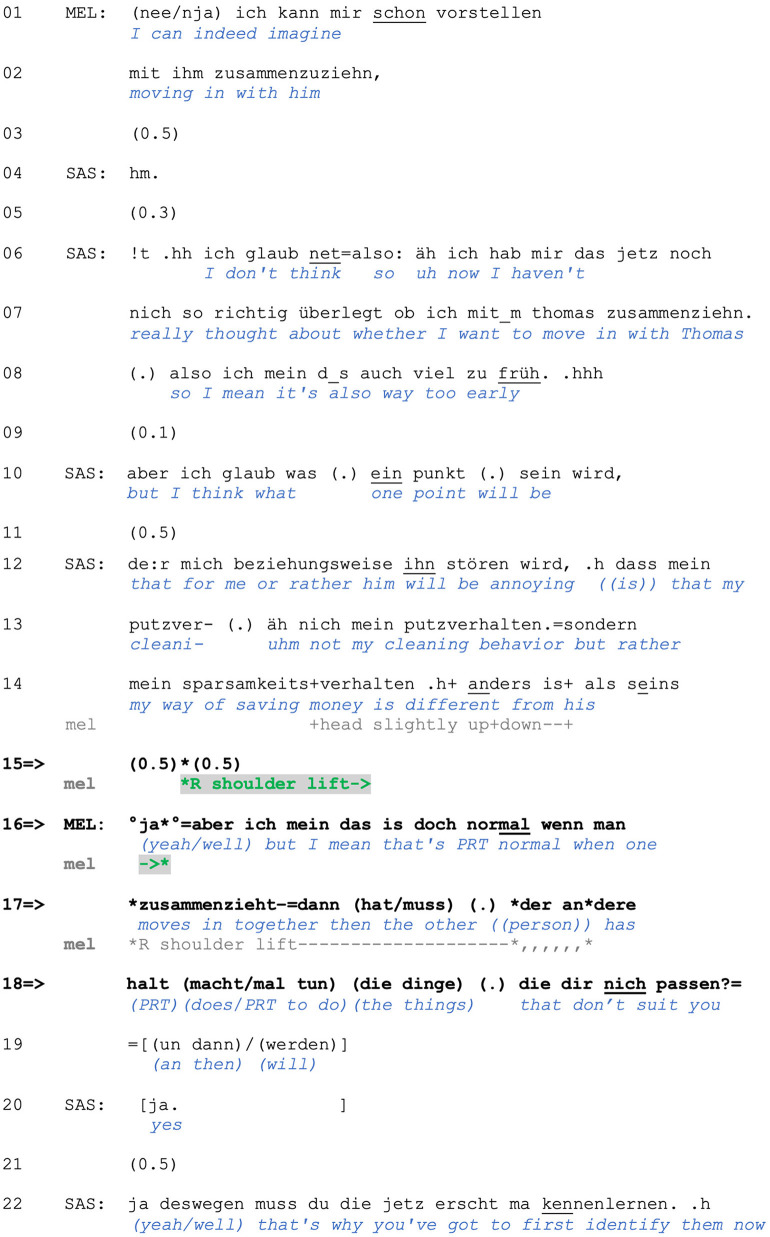
**Extract (3): FOLK_E_228_SE_01_T_01_c1310_das ist doch normal** https://bit.ly/3BPigaQ

The pause that emerges after Saskia's turn (line 15) can already project a dispreferred response to the problem, or hypothetical trouble, that Saskia describes. In line 16, Melanie responds verbally and assesses the situation Saskia just described as *normal*. With her assessment, which indexes problems with the prior action's terms with a turn-initial *ja* (“yes/well,” line 16; see Betz, 2017), Melanie casts Saskia's anticipated problems as a typical and expectable part of moving in together (rather than as out-of-the-ordinary, legitimate reason for *not* moving in together). In doing so, she pushes back against Saskia's displayed concerns. With the modal particle *doch*, she additionally appeals to common ground knowledge (Pittner, 2006) and thus to something that should be known to Saskia, which further contributes to rejecting the displayed problematicity of Saskia's concerns. All these features additionally contribute to Melanie's not just expressing her own subjective opinion on the matter but rather presenting her turn as an obvious fact known to, at least, the co-present interactants. In responding this way, Melanie curtails further development of this sequence into a troubles telling.

As in Extract (2), the shoulder lift in (3) precedes (and overlaps with the beginning of) a response that does not “go along” with the terms of the prior speaker's actions. However, while in Extract (2), a shoulder lift accompanied a *misaligning* response, it is used in (3) to project an upcoming (at least, partially) *disaffilaitive* response that rejects the relevance of Saskia's concerns. In prefacing her response with a shoulder lift, Melanie communicates resistance to engage in treating these concerns as expandable. The verbal turn that follows (lines 16–18) unpacks this positioning by framing Saskia's concerns as not out-of-the-ordinary but rather expectable (and therefore overblown here) and not expansion-worthy.

We also find *two-sided* shoulder lifts that occur in pre-beginnings of responsive turns. Extract (4), taken from the same fondue interaction as Extract (1), demonstrates this. Gero is telling Zoe and Norbert about his brother's first girlfriend, who their mother disliked. Norbert treats this information as difficult to reconcile with his own expectations of how Gero's mother would act. In response, Gero initially struggles to justify or explain his mother's behavior (lines 1–2). He then concedes that the situation was “weird” (line 6), which is supported by his description of the mother's reaction to the girlfriend's ultimate departure (line 9, see Pomerantz, 1986, on *extreme case formulations*). Zoe reacts with a short nasal laugh (line 11), while Norbert acknowledges the information with a minimal *okay* (Betz and Deppermann, 2021). After a pause, Zoe makes an assessment, *wie nett* (“how nice”). Accompanied by smiling and laughter, this assessment is intended as ironic and can be interpreted as casting either Gero's previous *formulation* of his mother's extreme reaction or the nature of *the reaction itself* (happiness) as insensitive or callous. In response, Gero raises his eyebrows, lifts both shoulders, opens his hands into a palm-up gesture, and states that he witnessed the situation first-hand (lines 14–15).

**Figure:**
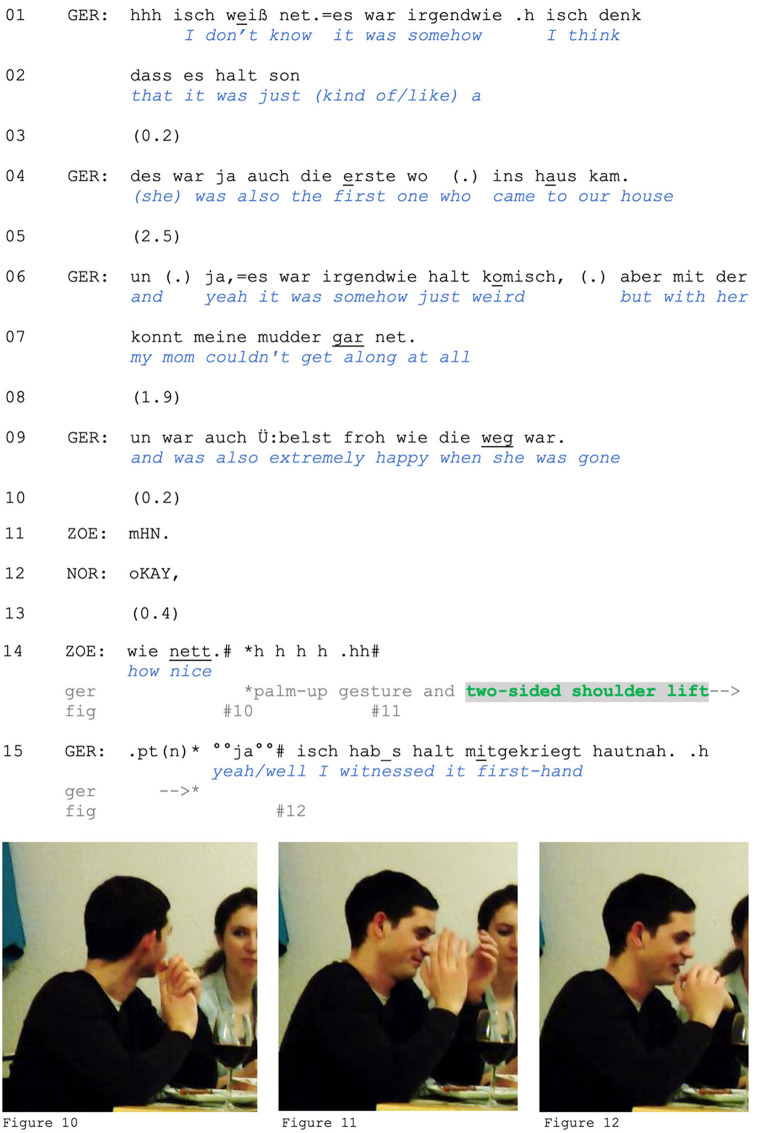
**Extract (4): FOLK_E_00293_SE_01_T_03_c234_wie_nett** https://bit.ly/40VNNSy

With his response in line 15, Gero provides evidence of his entitlement to produce the description in line 9. Unlike Extracts (2) and (3), a two-sided shoulder lift does not indicate the speaker's resistance to engage with the expectations set by the Other's action or activity. Instead, Gero's response accounts for his earlier turn (line 9), indicating that his knowledge is rooted in personal experience. In this context, the two-sided shoulder lift does not convey reluctance, unwillingness, or resistance to engage with Zoe's remark. Instead, it signals his inability to align with its implications [e.g., by recalibrating or reconsidering the stance he took previously, or by (co-)complaining about his mother] based on his experience. Gero's response to Zoe's negative, disapproving assessment frames his own prior statement as an objective recounting of events, rather than as a subjective or evaluative judgment. At the same time, with his turn in line 15, he positions himself as an observer with limited access to his mother's feelings, rather than as an active participant or evaluator of the situation. In doing so, he disclaims responsibility both for his mother's feelings and for his own description.

In this section, we have shown how shoulder lifts are used to project the type of the upcoming response and the stance taken in it. We have seen that one-sided shoulder lifts project responses that are in one way or another *uncooperative*, i.e., responses that do not “go along” with terms, expectations or agenda of the Other's prior action. In Extract (2), the recipient produces a misaligned and dispreferred response, namely a non-answer, which demonstrates a lack of willingness to engage with the prior search for an answer. In Extract (3), the recipient produces a disaffiliative response that rejects the problematicity of the Other's concerns and thus declines to affiliate with the prior speaker's stance. In both cases, speakers refuse to engage with, or commit to, the Other's initiated course of action, and the one-sided shoulder lift is crucial in conveying this stance. In contrast, the two-sided shoulder lifts in our data project responses in which speakers continue to orient themselves to the “social contract” between participants—namely, the moral responsibility of providing a cooperative response and re-establishing intersubjectivity (Extract 4). This is why two-sided shoulder lifts are of a more cooperative nature, as further sections will demonstrate. We have further shown how shoulder lifts are used to disclaim accountability, or responsibility, for what speakers are about to say by *distancing* from it, either by displaying a low epistemic (and deontic) stance and thus disclaiming the responsibility for the correctness of what is said (Extract 2 and 4) or by formulating the response as something self-evident (Extract 3).

### 5.2 Talk-accompanying uses of shoulder lifts

In addition to projecting a stance in a pre-beginning position, shoulder lifts can also index stance while accompanying verbal responses. Consider Extract (5) from a theoretical driving lesson. Before the extract begins, the instructor tells the student that she can opt to do license class C instead of C1. In lines 1–3, the instructor provides several advantages of this driving license category, which the student treats as news with *echt* (“really,” line 4; Gubina and Betz, 2021). In response, the instructor confirms (line 5) and restarts her turn from line 3, in which she frames the mentioned advantages as a reason for her not understanding people who choose to do C1 instead (lines 8–9). The student then initiates a verbal response and, in parallel, raises her eyebrows, turns down the corners of her mouth (Figure 14), shakes her head and produces a two-sided shoulder lift (line 10; Figures 14, 15).

**Figure:**
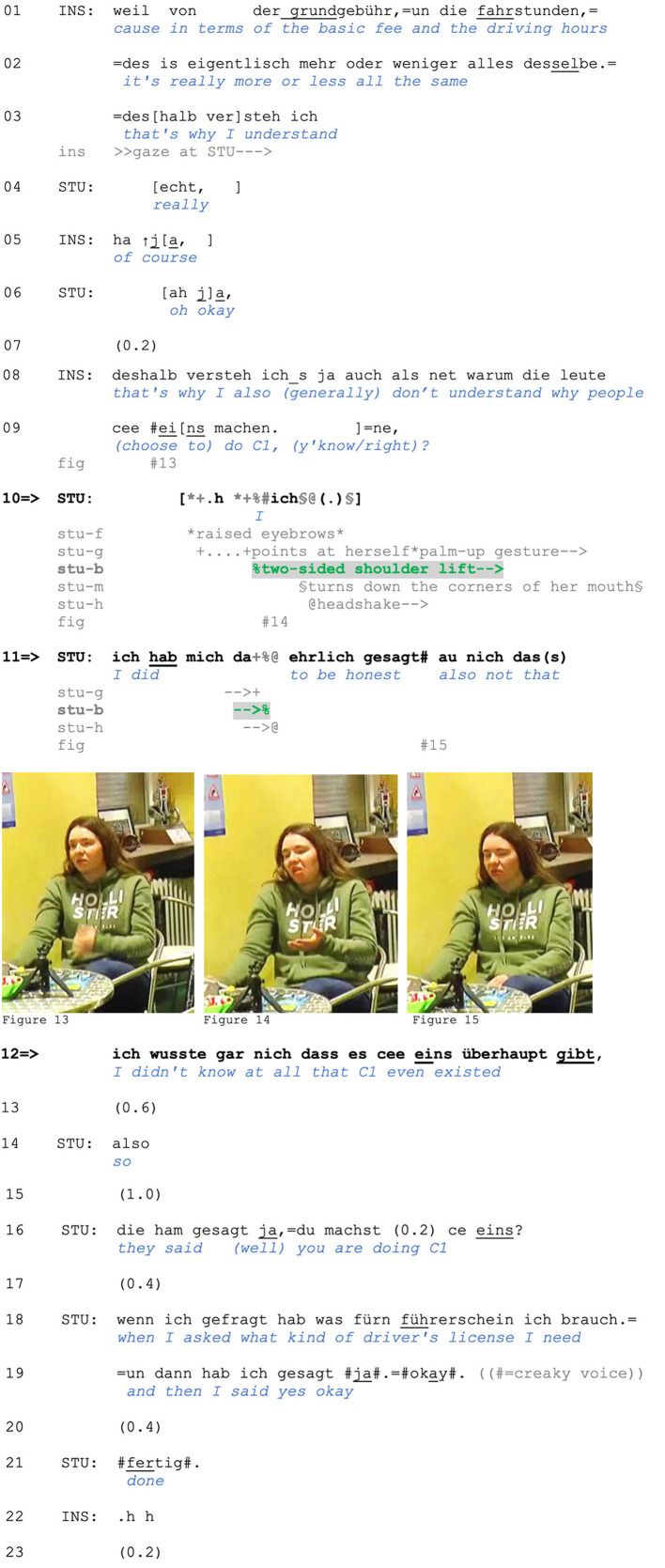
**Extract (5): FOLK_E_00348_SE_01_T_02_DF_01_c320_cee_eins** https://bit.ly/3A6oLpf

This is one of the prototypical environments in which two-sided shoulder lifts occur in our data. The two-sided lift, which in this case is at turn beginning, is produced in response to a criticism-implicative action by the prior speaker: Since the student is part of the group of “people who do C1” instead of the C driving license, the instructor's lack of understanding (lines 8–9) concerns the addressed student as well and can be seen as an account solicitation. The student's account is not only produced with multiple embodied displays but also with a hedge (*ehrlich gesagt* “to be honest”), orienting to the prior as a challenge through “speaking sincerely” (see Clayman, 2010). The two-sided shoulder lift prefaces an account that conveys the speaker's *inability* to fulfill the Other's expectations, which can be inferred from the negative epistemic marker that displays the student's lack of knowledge (line 12). Thus, the student is claiming an inability to make any other choice, which amounts to positioning herself as not responsible and accountable for her choices. This also becomes observable in the following sequential context, when the student is reporting that she was simply told what kind of driving license she needed. In doing so, she disclaims agency and ascribes responsibility for the decision to others (apparently, to her employer).

A similar use of a two-sided shoulder lift is found in the case we showed in Section 1. We are returning to the point at which Zoe produces a criticism-implicative turn regarding Gero's basis for judging his female colleagues. Zoe's turn does not explicitly assess Gero's behavior, although its design (a *dass* “that”-clause) projects a main clause containing Zoe's evaluation. Nevertheless, stating that Gero is assessing colleagues (of the other gender) only by their appearance (rather than their abilities, professional competence, character etc.) is criticism-implicative (and face-threatening) because he is thus positioned as superficial and/or unprofessional. Furthermore, Zoe uses the extreme case formulations *immer* (“always”) and *alle* (“all/everyone”) to frame his behavior as a pattern (see Pomerantz, 1986), which can serve as grounds for reprimanding. Zoe's turn in lines 10–11 can therefore be interpreted as an accusation. In response, Gero produces an account (line 13): He is not familiar with the intern's professional competence and thus lacks alternative grounds for judging her.

**Figure:**
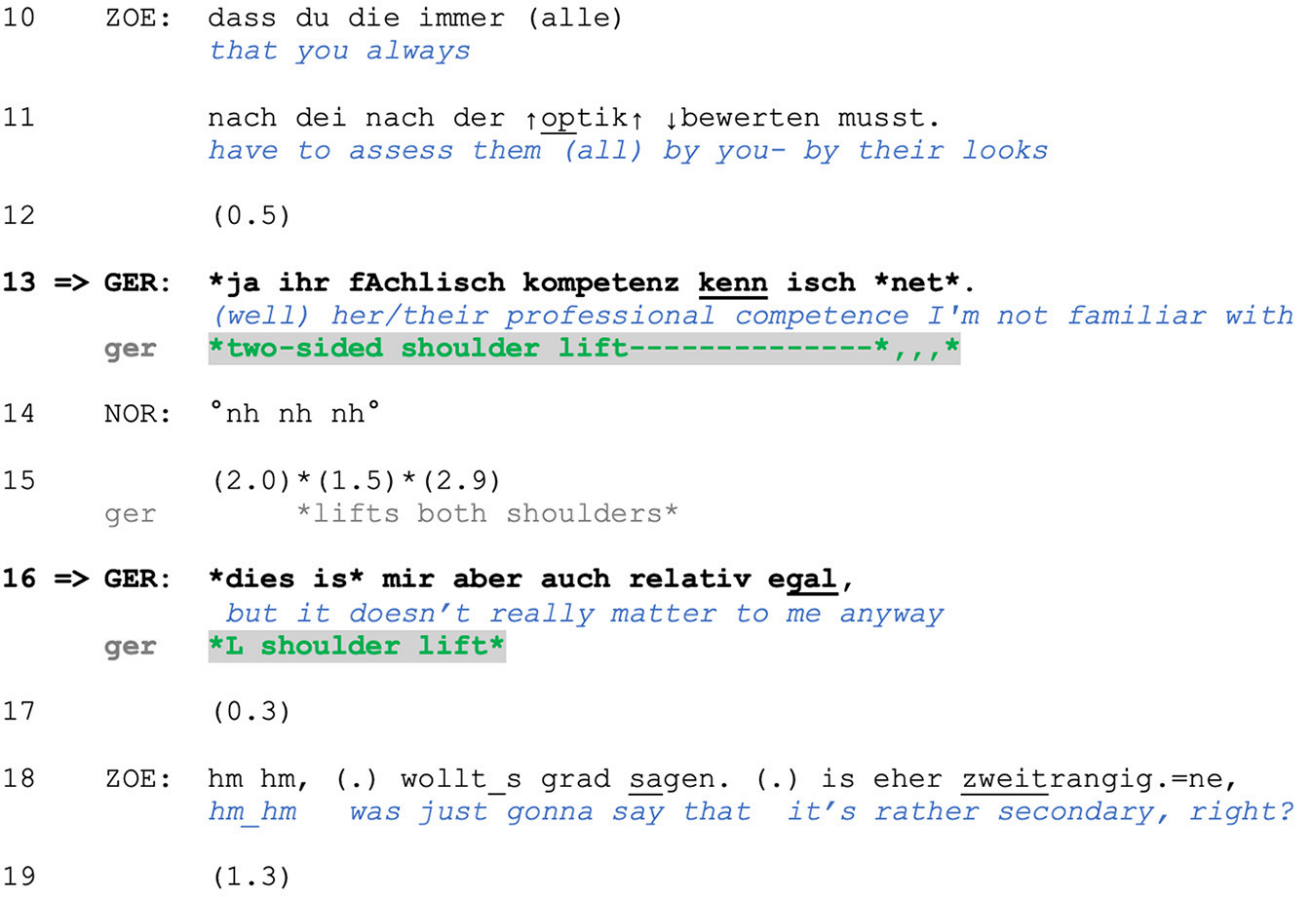
**Extract (6): FOLK_E_00293_SE_01_T_04_DF_01_c913_zweitrangig** http://bit.ly/3BS40hu

As in Extract (5), the two-sided shoulder lift accompanies a response to a criticism-implicative turn. Specifically, it is produced while the speaker accounts for his behavior by claiming inability to judge on other grounds due to Gero's lack of knowledge concerning the intern's professional competence (note also the turn-initial *ja* “yes/well,” which can index problems with the prior action's terms in responding; Betz, 2017). This claimed lack of epistemic grounds for making such judgments allows for Gero's positioning as not responsible or accountable for the criticized behavior and proposes sequence closure. In contrast, the second account he provides in line 16 does not claim an inability to meet the Other's expectations; it instead rejects the *relevance* of the prior point and thus of the basis for the criticism. We argue that this is a typical sequential environment for a *one-sided* shoulder lift, which in this case serves to display the speaker's lack of interest and, more broadly, to index resistance (rather than inability) to engage with the course of action initiated by the prior speaker.

This section has shown that responding speakers use both types of shoulder lifts to disclaim accountability and responsibility ascribed to them by prior speakers' actions. We have also shown that one-sided and two-sided shoulder lifts can convey different stances and thus lead to different outcomes in similar interactional environments. Two-sided shoulder lifts seem to mark the speakers' lack of *ability* to “go along,” or to further engage, with the Other's initiated or projectable course of action—due to a lack of knowledge (Extract 5, line 12; Extract 6, line 13). In contrast, one-sided shoulder lifts index some sort of *resistance* to “go along” with the Other's initiated or projectable course of action (Extract 6, line 16).

### 5.3 Shoulder lifts before possible turn completion

Shoulder lifts can also be produced at points at which (lexical-syntactic) completion is projectable. In such cases, they occur before a projected adjective or other assessing turn component in turns that embody an evaluative stance. The projected verbal component (and thus grammatical completion) is suspended and the (same) speaker produces a shoulder lift. Such shoulder lifts do not constitute completions of the incomplete turns but rather mark a stance toward the projected assessment.

We present three extracts to illustrate this use. The first comes from a job application training. The trainee (TRE) is describing to the trainer (TRR) and his assistant (AST) the quality of two recommendation letters he received from a past employer. *Zwei*(*er*) “two” (line 4) and *drei* “three” (line 7) denote overall grades, with “two” being a higher/better grade than “three” (and “one” being the best). After reporting the grade received for one letter, the trainee shifts to the description of *des andere* “the other one” (line 10). He had already reported earlier (not shown) that this report “read okay,” conceding that it was not of the highest quality by comparing it to the most desireable assessment, an “A+.” At line 12, he again initiates an assessment by uttering *(des andere) las sich* “(the other one) read.” The syntactic structure of the turn projects a somewhat positive assessment (e.g., *gut* “good,” *ganz gut* “pretty good”). However, instead of completing it, the trainee suspends the verbal turn in progress and produces a shoulder lift, a palm-up hand gesture (the latter in his lap and thus likely not visible to the trainer, see Figure 17), a slight mouth shrug, and a head shake (Figures 16–18).

**Figure:**
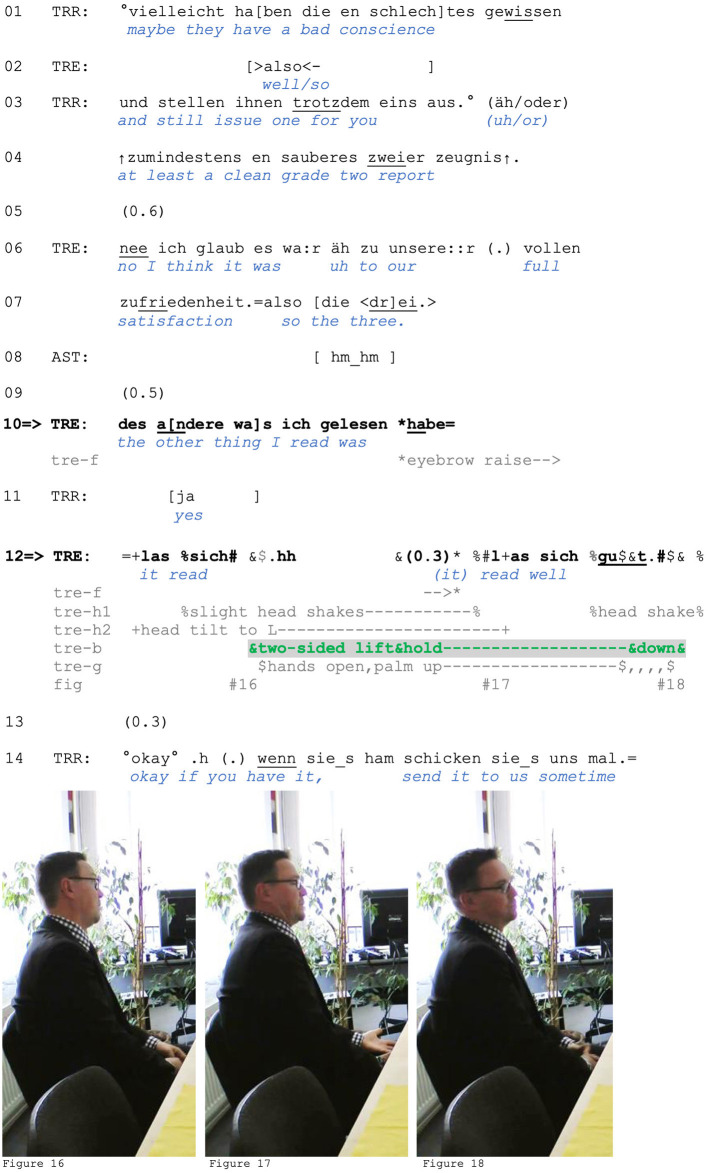
**Extract (7): FOLK_E_00173_SE_01_T_02_c669_las_sich_gut^4^** https://bit.ly/4gZ6paq

The two-sided shoulder lift is begun while the turn-in-progress (*las sich* “((it)) read”; line 12) is still incomplete. Specifically, the trainee's self-repair allows for the insertion of an embodied stance display. While the trainee is holding the gesture, he resumes his verbal turn and produces the projected assessment, *gut* (“good”). At its completion, he releases the shoulder lift. Interestingly, the shoulder does not constitute an embodied completion of the yet unfinished turn. Instead, it allows the speaker to display stance not with respect to a particular referent (the reference letter and its content) but rather with respect to his emerging positioning (Du Bois, 2007). The lift thus orients to the accountability for his assessment and specifically contributes to disarming, or disclaiming accountability: Since he did not get the best grade, a reference point he makes relevant in the prior context, this might imply that the reference letter is potentially critical or not that good, which contrasts with what he is saying in line 12. Furthermore, the trainee uses an impersonal format in formulating his assessment, which frames the assessment not as his personal opinion but rather as something that he is not accountable for. Thus, the turn design of his assessment allows him to distance himself from the claim of authorship and responsibility for the content of the reference letter being good.

We can also note that by marking the quality of the report beyond his control or influence, the trainee frames it as just being the way it is, which is why no further (sequential) expansion or elaboration is necessary. This is also what the headshake might be accomplishing in this turn, namely marking that “there is no need to discuss it” any further (Kendon, 2002, pp. 170–71). The trainer's reaction is responsive to this: His subsequent conduct suggests that he hears the trainee as communicating that he has nothing to add beyond what the trainee has arrived at with *gut*. With *okay*, the trainer accepts the trainee's description and moves to next steps (Mondada and Sorjonen, 2021): making arrangements for obtaining a copy of the letter in question (line 14).

However, not only the quality of the report, but also the *actual nature of the content* of the report is treated as beyond the trainee's control.^5^ Given that in this professional context, reference letters are expected to avoid explicitly negative phrasing, the trainee may be understood to express awareness that the way in which his reference letter “reads” does not necessarily reflect the actual evaluation expressed by it. In view of the prior context not included here, the shoulder lift can be seen as a resource for disclaiming expertise in reference letters, and ascribing (relatively more) expertise to the trainer, thus leaving him with the responsibility to ascertain the actual upshot of the reference letter. The request in line 14 may reflect that, having been designated the expert role, the trainer now knows he is responsible for reading the report himself to get an accurate picture of its contents, and he thus asks for a copy.

Recipients of such (syntactically) incomplete turns with shoulder lifts before a possible turn completion orient to those shoulder lifts as projecting a particular stance in an embodied way. This is visible in instances in which others treat the verbally incomplete turn as *complete*, for example, by responding with an agreeing second assessment. Extract (8), which comes from a theoretical driving lesson, illustrates this. The instructor is explaining the “BF 17” program for accompanied driving and the requirements for drivers who accompany students under 18 (which is the legal driving age in Germany). In lines 1–2, she criticizes the guidelines for not being specific enough, and a student agrees (line 4). After elaborating on this jointly with the student (lines 6–17), the instructor begins formulating a summary assessment, which includes elements that downgrade its strength: *also des is halt_n bissen* “so that's just a bit” (line 19).

Similar to Extract (7), the speaker does not bring her turn to its projected syntactic completion, which would, in this case, require an item that completes a negative assessment (of the guidelines she is supposed to teach) and can serve as a gloss for the situation instructor and student just expanded on (e.g., an adjective such as *unklar* “unclear,” *vage* “vague” or *blöd* “stupid,” see line 4). Yet, in contrast to Extract (7), in which the trainee himself completes the turn, even before lowering his shoulders again, the shoulder lift in this case occurs in a designedly incomplete turn, or an aposiopesis (Imo, 2011). When the adjective (or other negative evaluative term) is due, the instructor suspends talk and produces a two-sided shoulder lift (Figures 19–21). The sequence now develops differently than in the previous extract: In this case, the student responds before the turn completion by the instructor, and her response displays her understanding of where the prior speaker was going: She produces a negative assessment and marks it as an agreement with the instructor's stance (see *auch* “too,” line 21). In addition to formulating an agreeing second assessment, she produces a head shake, which marks her (affiliative) disapproving stance (Kendon, 2002). As in Extract (7), the shoulder lift does not *complete* the syntactically incomplete verbal turn. It does not communicate an evaluation itself but rather a stance toward the *projected* assessment, that of the lack of necessity to go on record with the word. With the lift, she orients to accountability for her position: The projected assessment is framed as obvious (so obvious it does not need to be put in words; and avoiding putting it into words may also be motivated by her role as a driving instructor teaching the rules that are being assessed negatively here) and as thus not worthy of elaboration.

**Figure:**
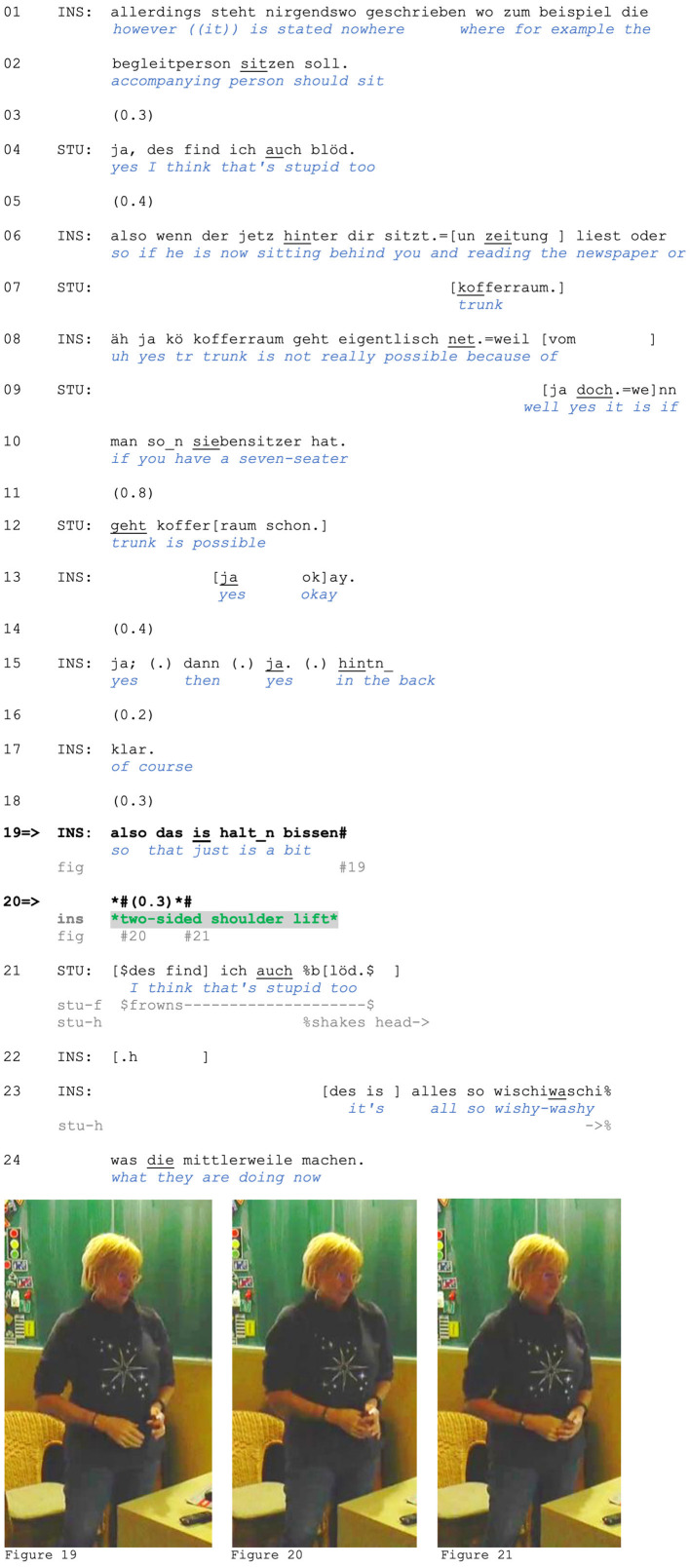
**Extract (8): FOLK_E_00348_SE_01_T_02_c153_wischi_waschi** https://bit.ly/4hfPwIT

Shoulder movements placed at points, at which (summary) assessments or upshots (e.g., with *also* “so” or *aber* “but”) are projected but not produced, can also be realized as one-sided lifts. With these, interactants emphasize the inescapability of the projected summary/conclusion as well as its obviousness. In Extract (9), the recognizability of what is left unsaid, and the grounds for treating it as obvious emerge from the larger sequential context. Elena and Norbert are newlyweds, and we join them in a conversation that takes place one week after their wedding. Earlier Elena had assessed time they spent with a friend on the preceding day as *schön* (“nice”), and she also reported talking to this friend on the phone and helping her identify guests on photos from the wedding. About 40 min later the exchange shown below happens. After a minimal embodied response by Norbert (line 13) to Elena's assessment *det sind so richtig coole freunde* (“they are just really great friends;” line 13), Elena continues with a specification, or a specifying account, a summary assessment (line 15), and a shoulder lift (line 17).

**Figure:**
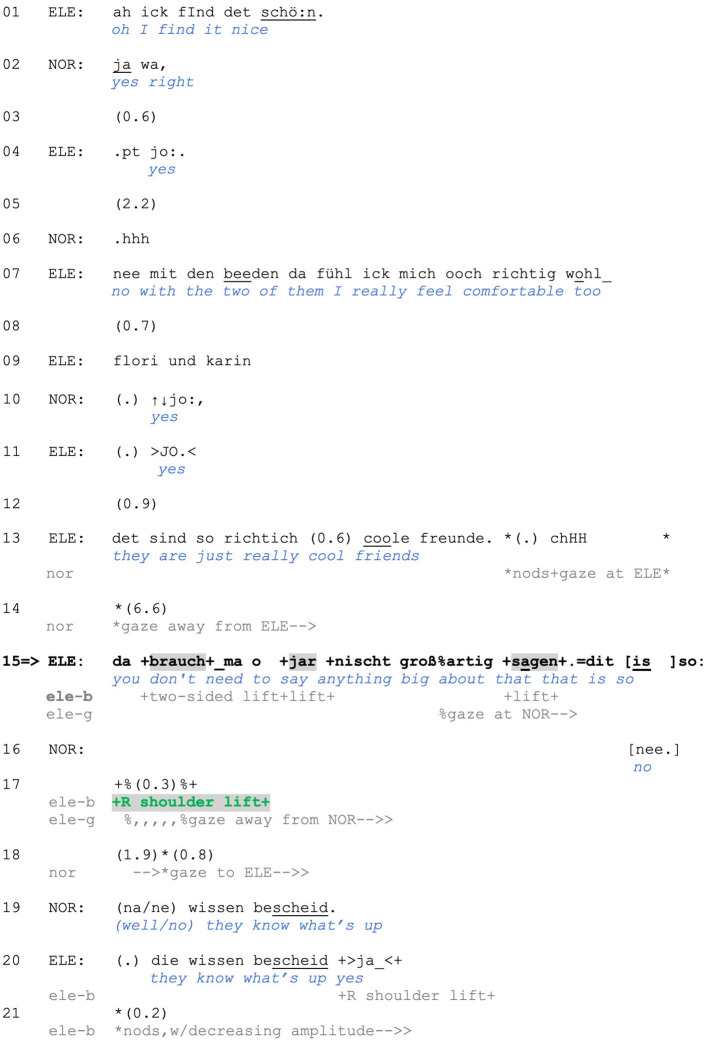
**Extract (9): FOLK_E_00039_SE_01_T_02_c1255_coole_freunde** https://bit.ly/4eG4aHo

In line 15, Elena describes a positive aspect of the friendship she just assessed positively (unspoken understanding), thus also accounting for her assessment. Furthermore, Elena's turn in line 15 is (at least partially) responsive to Norbert's lack of a response, given that her own prior turn (line 13) makes a second assessment or (dis)agreement expectable. In a second TCU in line 15 (and in overlap with Norbert's agreement with *nee* “no,” line 16) she begins to produce a projectable assessment of how she feels about the friendship with *dit is so:* “it's like/that is so” but does not produce the projected descriptor.^6^ Instead, she lifts her right shoulder. Similar to Extracts (7) and (8), rather than constituting an embodied completion of the projected assessment term (cf. Skogmyr Marian, 2021), a shoulder lift in this position conveys a particular stance toward the projected assessment, namely that it is obvious, or self-evident. This is also what Elena conveys with her first TCU in line 15, and it works toward disclaiming the speaker's own accountability and responsibility for the assessment (due to its self-evident nature). This obviousness of the assessment contributes to the meaning of the lacking necessity to expand any further, which is additionally highlighted by Elena's gaze shift away from Norbert (line 17). Norbert, however, now shifts his gaze *to* Elena (line 18) and offers a formulation that demonstrates his understanding of where Elena's turn was headed as well as agreement with her assessment (see Sacks, 1992, vol. I, pp. 146–47 on demonstrating vs. claiming understanding). Elena responds to this with a confirming repeat turn, indexing that Norbert has restated what she had already alluded to (see Schegloff, 1996b) and in her confirmation she produces another shoulder lift on the confirmation particle (see Section 4.1).

Extracts (7)–(9) have demonstrated how shoulder lifts contribute to stance management at points at which completion is projectable, more specifically before a projected adjective or other assessing turn component that display an evaluative stance. In such cases, the shoulder lift is produced when the projected grammatical completion is suspended. We have shown that such shoulder lifts *do not complete* the suspended turns but rather *mark a stance toward the projected* assessment. In particular, shoulder lifts can project an evaluative stance that can be problematic due to contrasting expectations and expertise (Extract 7) or due to the obviousness and self-evident nature of the upcoming and projected stance display (Extracts 8, 9). Furthermore, we have observed that shoulder lifts can occur either within a momentary, repair-like suspension before the speaker completes their turn (Extract 7), or with a designedly unfinished turn, after which no completion of the turn follows and another speaker takes over (Extracts 8, 9). This distinction arises because in Extracts 8 and 9, with aposiopesis, the turn end (i.e., what will complete the assessment) is clearly projectable for the interlocutor. In contrast, in Extract 7, the trainer cannot predict exactly what will follow.

### 5.4 Shoulder lifts in post-possible completion position

In Section 5.3, we presented shoulder lifts that occur before a possible turn completion. We have demonstrated how such lifts can project a stance toward an upcoming assessment or other evaluative descriptor before its production. We now turn to shoulder lifts that are produced in a post-possible completion position and show how these are regularly used as *post-completion* stance markers. “Post-possible completion” of a turn is a recurrent and systematic place for not just verbal turn extensions but also for elements that constitute “retrospective or retroactive alignments toward it, or consequences of it” (Schegloff, 1996a, p. 90), i.e., retrospectively taking a stance toward prior talk and what can be inferred from it. Such “post-completion stance markers” (ibid) can include grammaticalized elements, for example stance-marking particles in certain languages, and other linguistic and also non-vocal resources—“post-completion nodding, facial expressions (e.g., smiles or grimaces), shrugs, posture shifts, disclaimers (‘I dunno'), laugh tokens, coughs, exhalations and sighs, in-breaths, and I know not what else” (Schegloff, 1996a, p. 92; see also p. 121, note 37). In our collection, post-possible completion shoulder lifts are typically produced as two-sided lifts. In contrast, one-sided shoulder lifts are rare and similar to uses of shoulder lifts that precede and accompany talk, i.e., they communicate a stance of resistance rather than a stance of inability to do/say/add more (see Sections 5.1 and 5.2). In this section, we present two examples of two-sided lifts.

Our first case comes from the theoretical driving lesson that we already showed in Extract (5). In Extract (5), we focused on the student's account for choosing the driving class C1 instead of C: a lack of knowledge (lines 10–12). Note that the teacher slightly nods at this point (line 13) but continues gazing at the student. This can be interpreted as treating the student's account as incomplete/insufficient at this point and inviting further elaboration.

After the pause in line 13, the student produces *also* (“so”), which, similarly to the stand-alone *so* in English, “can be deployed to project an unstated upshot after a prior turn has been brought to a possible completion, and some silence begins to emerge, or after a recipient has produced some (minimal) uptake of that prior turn” (Raymond, 2004, p. 192; cf. Alm, 2015). Following this, the student performs two two-sided shoulder lifts while looking at the teacher (Figure 23), still refraining from continuing her turn. We argue that with these post-completion lifts, the student indexes her inability to further account or elaborate, thus disclaiming accountability for her choice of the driving lesson. While the student's interactional conduct in lines 13–15 can be interpreted as an attempt to invite sequence closure, the teacher's conduct does not align with this course of action, as evidenced by their lack of verbal response and continued gaze.

**Figure:**
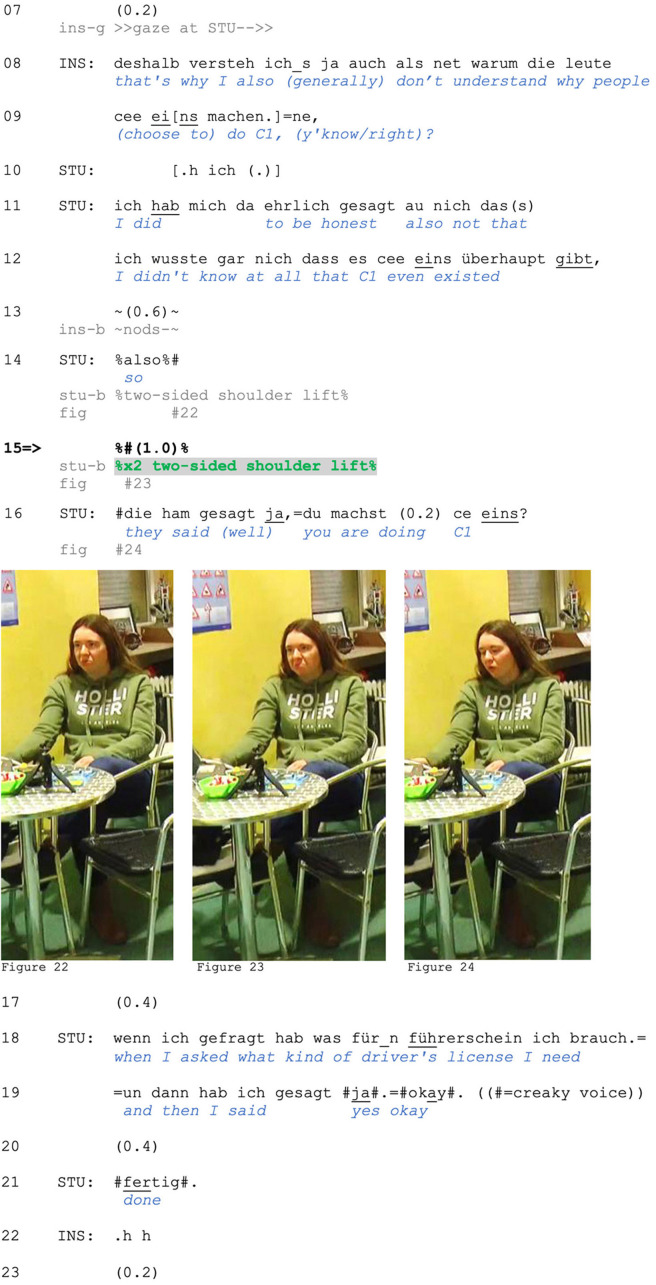
**Extract (10): FOLK_E_00348_SE_01_T_02_DF_01_c325_cee_eins** https://bit.ly/3A6oLpf

A similar case comes from a board game interaction. Christine, Renate, and Gabriele have just sat down; the fourth player Vanessa, who organized the game, set up the recording, and is also the most experienced player, is not yet seated. In her absence, Christine is distributing game pieces, and Renate is explaining aspects of the game to the novice player Gabriele (lines 7–10). In 12–15, Christine suggests that Gabriele change seats, which the latter resists in lines 16/18. Christine then proposes reseating herself (line 21). This is met with a rejection by Gabriele (*naa* “no,” line 23). The account that follows (reissued from line 16) suggests that the absent participant arranged the seating purposefully (*extra* “on purpose”), thus implying that the chair Christine proposes to fill was left empty by design. In formulating her account in this way, Gabriele ascribes the responsibility for the seating arrangement to Vanessa and thus disclaims responsibility and accountability for rejecting Christine's proposal. Instead of acknowledging Gabriele's account, Christine continues gazing at her. We are interested in Gabriele's bodily conduct at this moment (line 24).

After rejecting Christine's proposal and accounting for it (line 23), Gabriele's upper body moves slightly backward (see Figures 25, 26), while opening her arms and hands into a palm-up gesture (see Figures 28–30; Kendon, 2004; Müller, 2004). While Christine continues gazing at Gabriele and produces no response (lines 23–24), Gabriele lifts both shoulders (see Figures 29–31) and produces slight lateral head shakes before retracting the gesture (see Figure 8). This embodied conduct, which emerges simultaneously with Renate's confirmation in line 24, retrospectively contextualizes her verbal account in line 23: She conveys an inability to offer more than this account, which concerns the intentions of an absent third person. We can note that Gabriele's shoulder lift is done *after* the possible end of her verbal turn and in the face of Christine's continued gaze on her. This gaze may convey a continued expectation of further accounting for her rejection (Stivers and Rossano, 2010). With a post-completion two-shoulder lift, Gabriele communicates a particular stance of inability to further explicate, or elaborate, thus disclaiming accountability for the arranged seating and for not having more to say (which, as discussed above, she already accomplishes with her verbal account in line 23) and proposing sequence closure (see also Hoey, 2014: p. 184–5 on similar functions of post-completion sighs). In contrast to Extract (10), Christine here accepts this proposal by receipting Gabriele's response (line 25) and also performing an embodied shift to game objects.

**Figure:**
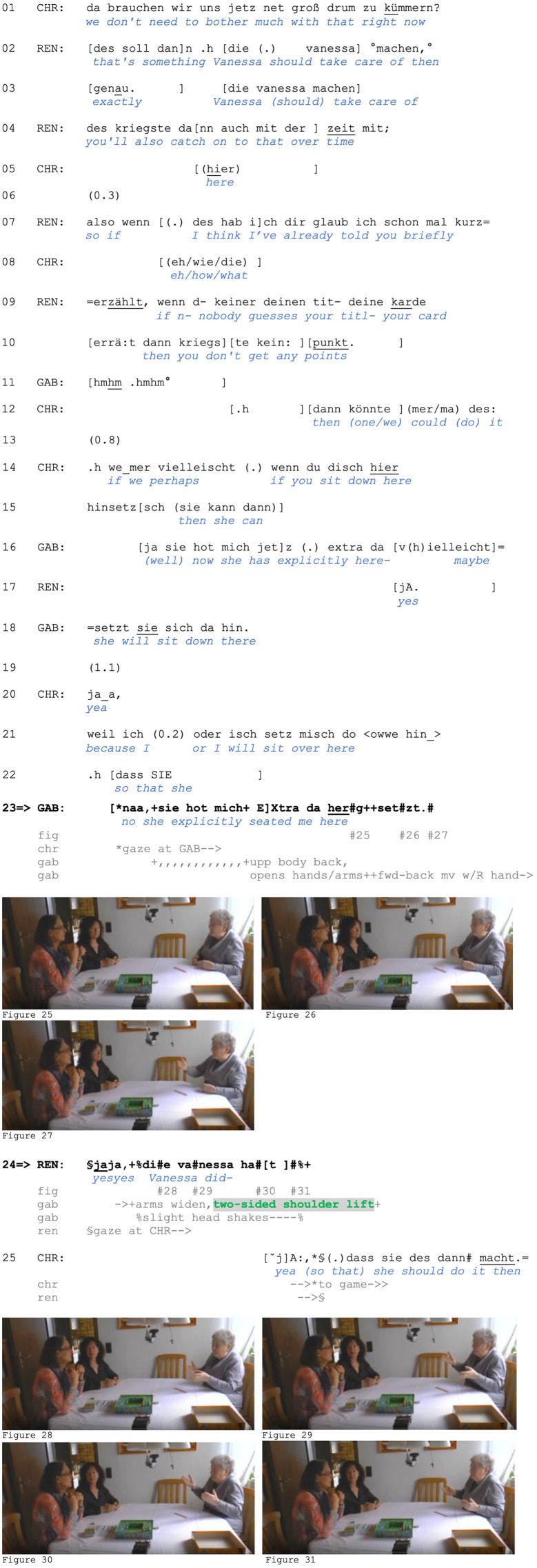
**Extract (11): FOLK_E_00357_SE_01_T_01_c64_extra_dahergesetzt** https://bit.ly/4h2vUaP

In this section, we have demonstrated how shoulder lifts are used in post-possible completion position. Such shoulder lifts, which in this position typically occur as two-sided shoulder movements, seem to push back against the Other's expectations of more to come (which are usually occasioned and displayed by continued gaze and/or focus of the Other toward the recipient) as well as reject further accountability. One-sided shoulder lifts, by contrast, can function as stance markers that can retrospectively frame assessments (e.g., downplay their reach or consequentiality in the here and now) and thus can help speakers manage face-threatening aspects of their prior action.

### 5.5 Shoulder lifts as stand-alone responses

In the previous sections we have seen how shoulder lifts are connected to (precede, co-occur, follow) talk that elaborates or restricts the movement's import. But shoulder lifts can also be used as stand-alone responses to convey resistance to another speaker's proposed course of action. In other words, shoulder lifts can be complete and coherent actions in themselves, as evidenced by participant orientations to them. In these uses, it is the ongoing course of action and the relevancies and expectations established by the prior turn that allow the shoulder lift to take on a particular meaning and convey a specific responsive action.

We have already seen (although not yet discussed) such a shoulder lift in our initial extract, to which we now return. Recall that Zoe had produced a criticism-implicative turn, targeting Gero's criteria for judging women. In response to what can be heard as an accusation, Gero produces an account (line 13, see detailed analysis of this in Section 5.2). He claims a lack of knowledge (i.e., an inability to use a better/more acceptable basis for his judgement) as a defense. Zoe does not respond to this; a silence emerges, in which Zoe continues eating and then shifts her gaze to Gero. Following this, Gero lifts both shoulders (line 15).

**Figure:**
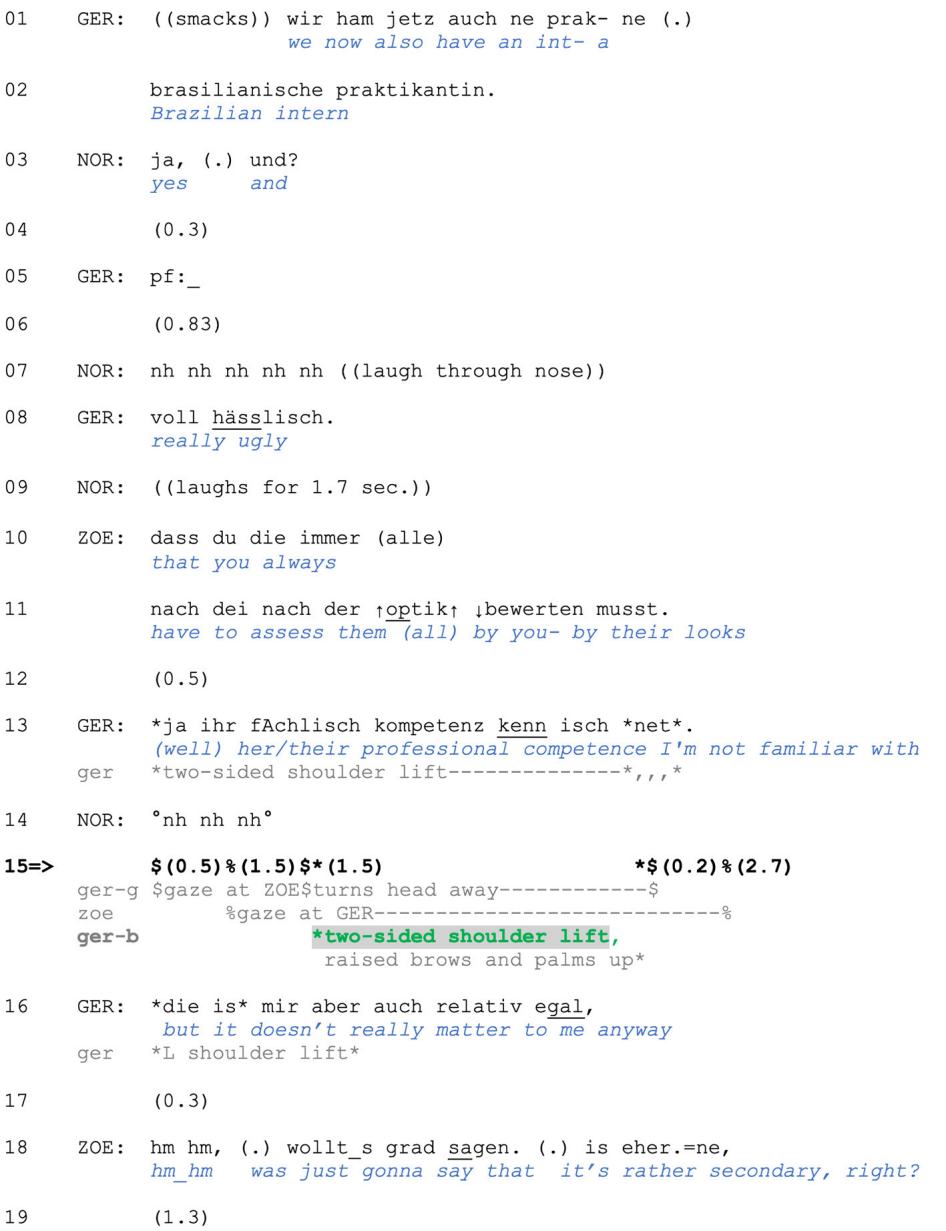
**Extract (12): FOLK_E_00293_SE_01_T_04_DF_01_c913_zweitrangig** http://bit.ly/3BS40hu

Gero's two-sided shoulder lift is produced after he has finished an account (line 13). It is, however, not designed as a post-completion stance marker. Note that Zoe shifts her gaze to Gero in line 15, which might be indexing the continued relevance of accounting. Gero seems to respond to this “requesting” gaze with a shoulder lift. This embodied response disclaims (further) accountability and retrospectively frames his verbal account as inescapable and non-expandable. He has already produced an account for the criticized behavior and (thus) has nothing else to add in his defense.

In Extract (13), the responding speaker conveys a refusal/declining of the opportunity to engage with a proposed action (assessing) via a shoulder lift, and it is oriented to as such. Ferdinand and Isabell are driving on the highway. Isabell has just checked the projected arrival time and assessed it negatively (i.e., as later than expected). Line 1 could be proposing a (non-serious) solution to this “problem”: Driving faster will get them there earlier. In line 4, Ferdinand shifts his gaze to where one of the cameras is mounted, and in line 5, he formulates a noticing (while Isabell seems to be reading a highway sign announcing a landmark or upcoming regional attraction: *rock pop museum*, lines 6–7). Isabell then shifts her gaze to the same spot and confirms Ferdinand's observation (line 7). In overlap, Ferdinand offers a revised understanding of an action they did earlier (line 8), which can be seen as troubleshooting, and he assesses the whole situation as *nervig* “annoying” (line 9). Isabell responds with a shoulder lift (line 10, Figures 33, 34).

**Figure:**
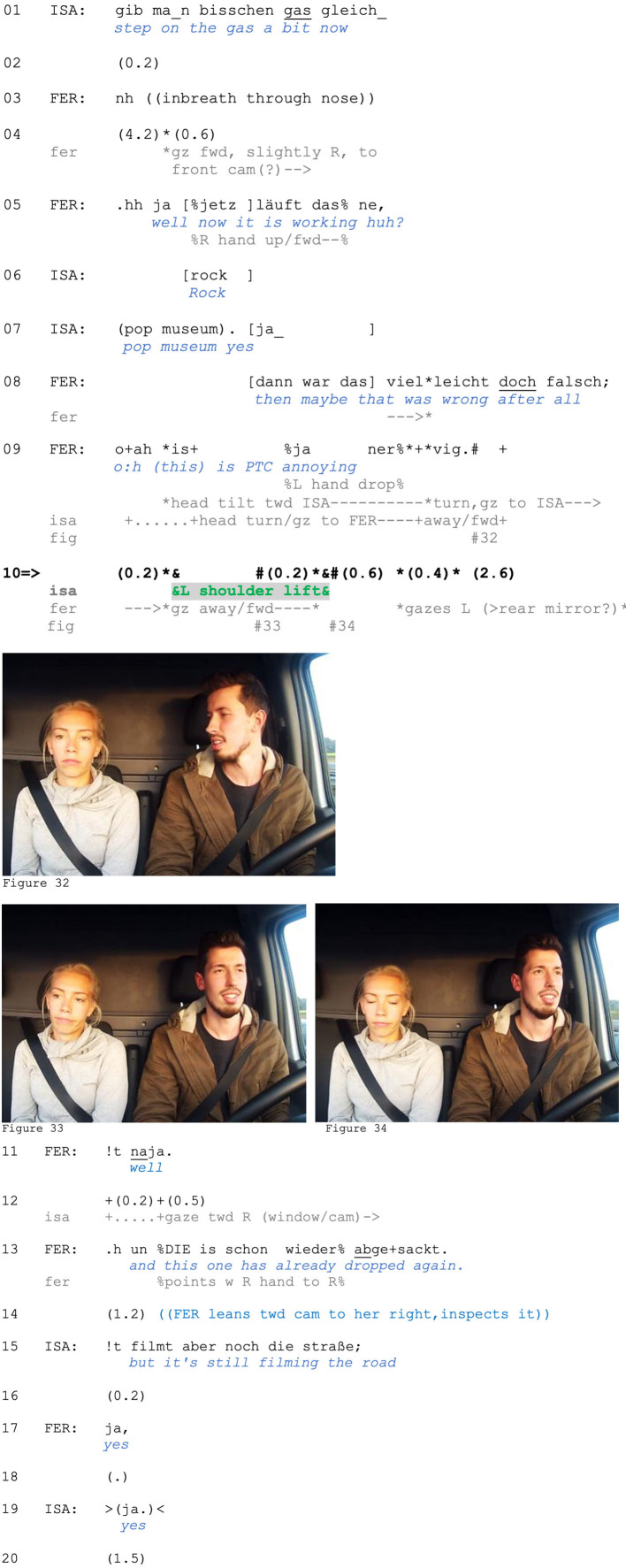
**Extract (13): FOLK_E_301_SE_01_T_02_c116_is_ja_nervich** https://tinyurl.com/4x8jacx9

By expressing disappointment or regret in line 9 (notably with *oah*, Couper-Kuhlen, 2009; Golato, 2012), Ferdinand not only invites a second assessment but also offers an opportunity for Isabell to co-complain, or, at least, to affiliate with him. After Ferdinand directs his gaze to Isabell (mobilizing response; see Figure 32), Isabell produces a one-sided shoulder lift (with a head tilt and mouth shrug) as a response. Although Ferdinand is shifting his gaze away from Isabell as she produces the lift, her movement—done with her left shoulder, which is close to him—is likely still perceivable to Ferdinand. He responds with disengagement (gaze to left car mirror, line 10), a click, which can close down a current topic (Ogden, 2013), and a stand-alone *naja* (“oh well,” line 11), which marks readiness to shift the topic while passing on the opportunity to expand or nominate a next one (Golato, 2018, pp. 420–22). Ferdinand then moves on to an observation about the other camera, and this is taken up by Isabell.

This extract provides sequential evidence for a co-participant's understanding/treatment of a stand-alone shoulder lift in responsive position. Isabell does not just decline to offer a verbal response but takes a particular stance toward Ferdinand's prior action: She declines to engage in the line of action Ferdinand offered in line 9 (co-complaining) by treating it as not worthy of expansion (or of any verbal response at all). Thus, like Extract (12), the shoulder lift here functions as a sequence exit device, which is oriented to by Ferdinand with a topic shift.

## 6 Conclusion and discussion

This study has focused on the use of one-sided and two-sided shoulder lifts in German talk-in-interaction and the interactional work they can accomplish in various turn positions. Focusing on broadly responsive uses, we have demonstrated how shoulder lifts are used in four major turn positions in our data, namely in pre-beginnings of the turn, accompanying the turn, at points at which completion is projectable and in a post-possible completion position. Shoulder lifts that occur in pre-beginnings typically project the uncooperative (misaligning or disaffiliative) nature of the upcoming action. Talk-accompanying shoulder lifts usually contribute to the uncooperative stance embodied by the action of the verbal turn. Shoulder lifts that occur before a possible turn-completion typically pre-empt projected evaluative terms and thus show speakers' orientation to the possible problematicity or non-necessity of producing the turn completion verbally. Finally, shoulder lifts in post-possible completion position mark the completeness and non-expandability of what was said before and thus push back against possible expectations of the Other for more to come. To summarize, our study elaborates the contribution of precise sequential placement within TCU and beyond to the function of a bodily resource or movement (Schegloff, 1996a). Thus, these results contribute to recent research analyzing the positionally-sensitive nature of bodily resources and sound objects in talk-in-interaction (e.g., Clift, 2021; Ford et al., 2012; Hoey, 2014, 2020; Ogden, 2013).

We have also demonstrated that one-sided and two-sided shoulder lifts seem to contribute to accomplishing different interactional work. Specifically, two-sided shoulder lifts regularly mark speakers' lack of *ability* to “go along,” or to further engage with, the Other's initiated or projectable course of action, often due to a lack of knowledge. Hence, such shoulder lifts could be seen as indexing low epistemic stance. In contrast, one-sided shoulder lifts tend to mark resistance to “go along,” or to engage with, the Other's initiated or projectable course of action. In doing so, they aim to curtail the Other's line of action. Thus, although both kinds of shoulder lifts could be seen as uncooperative in some way, two-sided shoulder lifts still work toward re-establishing intersubjectivity and thus seem to be *relatively more cooperative* than one-sided shoulder movements.

Our research contributes to a growing body of work that focuses on the *division of labor* among variants of specific embodied resources (e.g., one- vs. two-sided), or how the shape/quality of a resource (e.g., small vs. conspicuous movement) can affect the functional spectrum of that resource (cf. Helmer et al., 2021 on downward vs. upward head nods; Hömke et al., 2017 on short vs. long blinks; Debras and Cienki, 2012 on head tilt left vs. right). This clearly demonstrates “order at all points” (Sacks, 1984, 1992) in the situatedness of various resources in interaction, specifically in the meaning that interactants derive from how talk, bodily resources, and sequential placement shape and particularize meaning together. As our analyses show, shoulder lifts also systematically co-occur with other body movements, such as head tilts, eyebrow raises, or palm-up orientations of the hands. Future research is needed in order to clarify to what extent these other movements matter for the interpretation of the shoulder lifts or are perhaps typical of particular uses of shoulder lifts.

We have also been able to determine the main job of shoulder lifts across different uses and contexts, i.e., a possible *context-free meaning*, namely indexing disclaiming accountability or responsibility for the projected or produced turn-at-talk. Our results show that there are different types of accountability that can be indexed as reduced with shoulder lifts: First, in cases in which they are produced in response to account solicitations or challenges, shoulder lifts work toward disclaiming *moral* accountability (see Extract 4, 5, or 6). Second, lifts can be used to push back against being made accountable for (not) producing a specific response and (not) following the normative expectations set by the Other's prior action (e.g., Extract 2, 3, 11, or 12). Finally, shoulder lifts can function in an anticipatory manner: Speakers can produce them to *pre-empt* something that might make them accountable (e.g., Extract 7, 8). Thus, this research presents new findings concerning the role of embodied resources in managing accountability and responsibility in talk-in-interaction (see Heller, 2018; Robinson, 2016).

Our findings also contribute to the growing scholarship on embodied resources in stance-taking in talk-in-interaction. In particular, we have shown that in contrast to many other bodily movements that can embody particular stances in social interaction (like iconic gestures or specific facial expressions, e.g., Kendon, 2004; Skogmyr Marian, 2021; Streeck, 2009), shoulder lifts do not take a stance toward the referent of their verbal turn nor toward the referent of the Other's prior turn. We have further shown that shoulder lifts are not connected with negative or positive stances, or connotations. Instead, shoulder lifts operate on another level of stance—stance toward what the speakers themselves are saying (or are about to say or just said) as self-evident, inescapable, obvious, and (thus) not further expandable or negotiable.

While one-sided and two-sided shoulder lifts in German seem to accomplish different interactional work, more cross-cultural and cross-linguistic research is needed to examine whether a similar distinction can be found in other cultures and languages as well. Furthermore, as we mentioned in Section 4, we excluded cases of shoulder lifts occurring in multi-unit turns from the current analysis. Thus, it would be important to examine such occurrences of shoulder lifts from a micro-analytic perspective in future studies. Finally, our work helps specify the “variety of meanings” (Debras, 2017, p. 29) for components of the “shrug,” thus expanding our understanding of the context-specific use of the body for action in *talk-and-bodily-conduct-in-interaction*. Future research could explore the interactional work that can be accomplished with other components of what is understood as “shrugs,” such as mouth shrugs, head shakes, or head tilts. This would enable us to pinpoint the specific functions performed by different prototypical components and to better understand how each of them contributes to “shrugs” as complex ensembles in social interaction.

## Data Availability

The data extracts presented in this study are from the Database of Spoken German, Forschungs- und Lehrkorpus gesprochenes Deutsch (FOLK) and can be found online at dgd.ids-mannheim.de.

## Footnotes

^1^It should be noted that such studies primarily concentrate on affective, or emotional, stance (but see, e.g., Cekaite, 2010; Deppermann and Gubina, 2021; Goodwin and Cekaite, 2013; Heller, 2018; Schoonjans, 2018 on bodily and paralinguistic means for displaying epistemic and deontic stance).

^2^The corpus is hosted at the *Leibniz Institute for the German Language* and is accessible to scholars after registering at http://dgd.ids-mannheim.de. For a detailed description of the German FOLK-corpus, see Schmidt (2016).

^3^The beginning of Extract (4) shows such shoulder lifts. They are produced by the the driving instructor (see line 3).

^4^Abbreviations used in the focus lines: Tre-f: face; Tre-h1: head1; Tre-h2: head 2; Tre-b: body; Tre-g: gesture.

^5^We thank an anonymous reviewer for prompting us to make this dimension more explicit.

^6^The incompleteness of Elena's TCU could also be accounted for by Norbert's early affiliative response *nee* (“no”, line 17).
